# Efficient Model-Based Anthropometry under Clothing Using Low-Cost Depth Sensors

**DOI:** 10.3390/s24051350

**Published:** 2024-02-20

**Authors:** Byoung-Keon D. Park, Hayoung Jung, Sheila M. Ebert, Brian D. Corner, Matthew P. Reed

**Affiliations:** 1 Biosciences Group, University of Michigan Transportation Research Institute, Ann Arbor, MI 48109, USA; hayjung@umich.edu (H.J.); ebertshe@umich.edu (S.M.E.); mreed@umich.edu (M.P.R.); 2 Corner3d LLC, Bedford, VA 24523, USA; bdcorner3d@gmail.com

**Keywords:** 3D anthropometry, body dimension measurement, ANSUR, whole-body scanning, inscribed fit, body characterization, clothed scan measurement

## Abstract

Measuring human body dimensions is critical for many engineering and product design domains. Nonetheless, acquiring body dimension data for populations using typical anthropometric methods poses challenges due to the time-consuming nature of manual methods. The measurement process for three-dimensional (3D) whole-body scanning can be much faster, but 3D scanning typically requires subjects to change into tight-fitting clothing, which increases time and cost and introduces privacy concerns. To address these and other issues in current anthropometry techniques, a measurement system was developed based on portable, low-cost depth cameras. Point-cloud data from the sensors are fit using a model-based method, Inscribed Fitting, which finds the most likely body shape in the statistical body shape space and providing accurate estimates of body characteristics. To evaluate the system, 144 young adults were measured manually and with two levels of military ensembles using the system. The results showed that the prediction accuracy for the clothed scans remained at a similar level to the accuracy for the minimally clad scans. This approach will enable rapid measurement of clothed populations with reduced time compared to manual and typical scan-based methods.

## 1. Introduction

Three-dimensional (3D) surface measurement has become a central component of anthropometric surveys (Bartol et al., 2021 [1]; Bonin et al., 2022 [2]; Gordon et al., 2013 [3]; Gordon et al., 2014 [4]; Goto et al., 2019 [5]; Lu and Wang, 2008 [6]; Park et al., 2014 [7], Robinette et al., 2002 [8], Tsoli et al., 2014 [9]). Modern surface scanning equipment can accurately capture the shape of the surface of the body in a fraction of a second. However, the practical aspects of conducting 3D scanning surveys have changed little in the past 25 years. In particular, participants are required to change into close-fitting garb that minimizes the clothing effects on the subsequent scan. This clothing ensemble must be provided, along with suitable privacy for changing. The consequence is that several seconds of scanning can require 10 min or more of preparation for each participant and considerable resources.

Recently, studies on fitting virtual avatars, such as statistical human models, to clothed body scans have attempted to capture body shapes under clothing (Guan et al., 2010 [10]; Hasler et al., 2009 [11]; Hu et al., 2021 [12]; Pishchulin et al., 2017 [13]; Yang et al., 2016 [14]; Lu et al., 2021 [15]). Bălan and Black (2008) [16] presented a model-based body shape estimate system that finds a maximal silhouette-consistent shape to estimate body shape from a number of 2D images. Hasler et al. (2009) [11] developed a technique using a statistical body shape model and an iterative closest point (ICP)-based Laplacian mesh deformation approach to predict the body shape of dressed humans. Guan et al. (2010) [10] presented a method focusing on two-dimensional models for modeling clothing deformations on the body. Zhang et al. (2017) [17] estimated the inner body shape in various postures while recovering surface details. These studies commonly require high computational resources to solve expensive optimization problems in order to estimate body shapes. Also, they focused on estimating visually realistic 3D body shapes rather than measuring body dimensions; thus, the results were not validated against actual dimension measurements. Hu et al. (2021) [12] proposed a method that applied machine learning to predict undressed body shapes from dressed human scans by generating a dataset of synthetically combined dressed human and undressed ground truth body shapes.

In a previous study, the study team introduced an efficient underlying body shape estimation method called the Inscribed Fitting (IF) method (Park et al., 2016 [18]), based on a rapid model-based fitting technique (Park et al., 2014 [7]). This IF method uses an iterative process to estimate the body shape from clothed scan data, based on the observation that the correct body shape is well estimated by the largest body shape that does not protrude through the clothing. The fitting is performed by maximizing a goodness-of-fit metric through the choice of shape factor scores, where the shape factors are the principal components of the body shape model. The model output includes a set of predicted anthropometric dimensions and anatomical body landmark data, as well as a realistic body shape.

The main objective of the current study was to develop a portable model-based anthropometry system to obtain body dimensions of individuals by capturing 3D body shapes of clothed individuals using low-cost depth sensors. Three low-cost depth sensors (Microsoft Kinect V2) were utilized to capture the 3D body shape in a fraction of a second. The system consists of two parts: (1) a data collection component to operate the sensors and process gathered depth images over a network, and (2) an anthropometry component to fit a statistical body shape model to the scans and estimate the most likely body shape along with a set of body dimensions and landmark locations.

A total of 144 adults were recruited to quantify the measurement accuracy of the current system against a whole-body laser scanning system. The quantitative accuracy of the system was evaluated using data from two levels of clothing. The quality of the inscribed fits was evaluated through quantitative comparison to minimally clad laser scans from the population as well as by comparing the predicted standard anthropometric dimensions with manual measurements.

## 2. Materials and Methods

### 2.1. Model-Based Anthropometry System

**Figure 1:**
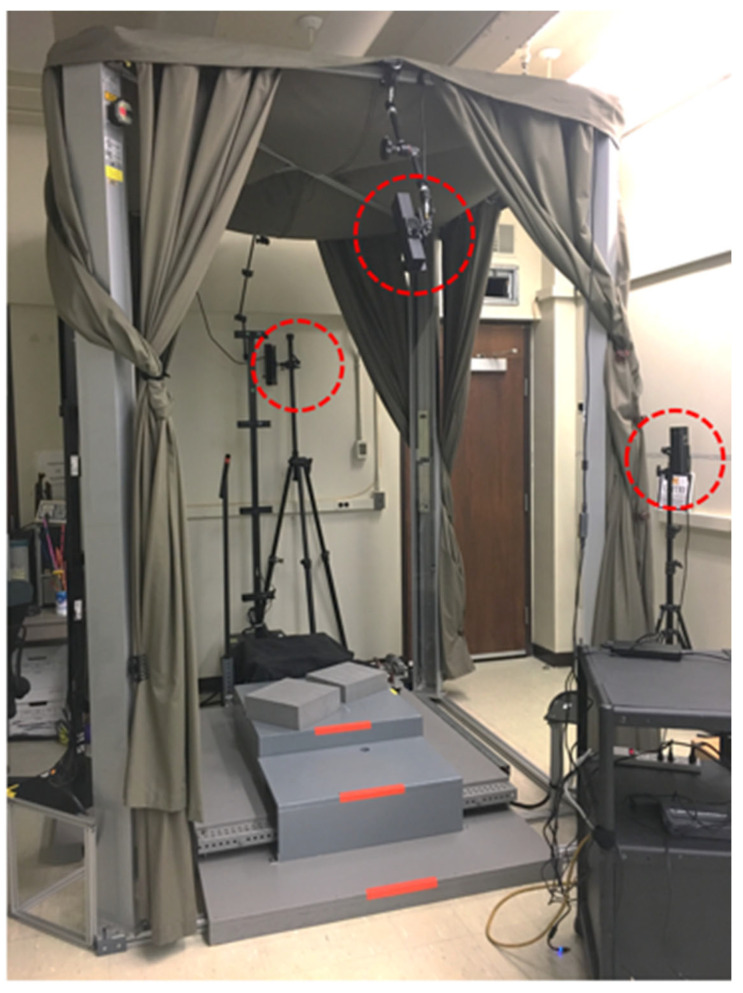
Kinect V2 sensors (red circles) with a whole-body laser scanner (VITUS XXL).

The system hardware system consists of three Microsoft Kinect V2 sensors aimed at the front, back, and side of the participant. The high bandwidth of the sensor necessitates a dedicated computer for each sensor. Figure 1 shows the sensors positioned for the current study. The Kinect sensors were installed on the structure of a whole-body laser scanner (VITUS XXL) to enable near-simultaneous imaging of the participant by both systems. Kinect sensors placed on the front and rear sides are used for overall geometry capture, and sensors placed on the side are used for alignment of the captured geometry data.

Data collection software consists of a server program and a client program. The server program sends a signal to clients and gathers the scan data over a network while synchronizing the frames from each sensor. The client program utilizes the Kinect API to capture the depth and color data and combine them when a capture signal is received from the server. The client reduces the noise level by analyzing multiple depth images obtained over 150 ms (Park et al., 2014 [7]) and compresses the combined depth+color data to facilitate smooth streaming over the wireless network. The server program aligns the captured scans using the iterative closest point technique and stores the transformation information to merge the scans from the sensors. The height of the floor in the measurement coordinate system is stored in this step.

The scanning posture was standardized according to the MCANSUR report (US Marine Corps Anthropometric Survey, Gordon et al., 2013 [3]) as follows:

- The participant stands on the scanner platform with his or her feet on “footprints” painted on the platform. The footprints are positioned 30 cm apart.
- The participant stands erect with the weight distributed equally on both feet.
- The arms are straight and held away (30°) from the body with fists clenched.
- The participant looks straight ahead.
- The participant breathes normally and stands relaxed without flexing his or her muscles.

**Figure 2:**
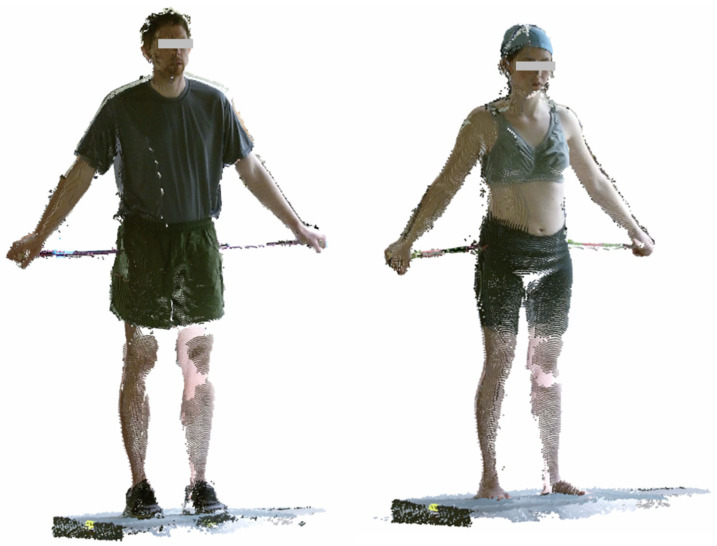
Examples of processed scans in A-pose from the Kinect sensors. From the left, physical training (PT) and scan wear (SW) conditions.

Figure 2 shows examples of the processed Kinect scans in this standardized standing posture. 

### 2.2. Statistical Body Shape Model

This study used separate male and female statistical body shape models (SBSMs) that were based on 1224 male and 591 female scans from the MCANSUR survey. These standing scans were homologized using a template fitting method (Park and Reed, 2015 [19]) to standardize the mesh structure across the scans. The SBSMs were built by conducting a principal component analysis of the standardized scan vertex coordinates along with 74 body landmarks, 19 joint locations, and 136 manual anthropometric measurements (Park and Reed, 2015 [19]). A total of 60 principal components were retained for each model to represent 99.7% of the variance in the body shape, landmark locations, and anthropometric dimensions.

### 2.3. Enhancement of Inscribed Fitting (IF) Method

The IF method was developed to estimate the body shape underlying clothing (Park et al., 2016 [18]). The method is based on a rapid model-based fitting method (Park et al., 2014 [7]) that finds a set of body shape factors (principal component scores, PCs) that generates the closest shape to the target shape in a statistical body shape space. Briefly, the IF method finds the most feasible body shape from a clothed scan by assuming that the correct body shape is the largest body shape that does not protrude through the clothing. This largest body shape is found by adjusting the PCs iteratively to push the initially fitted model’s vertices to lie just inside the target surface.

**Figure 3:**
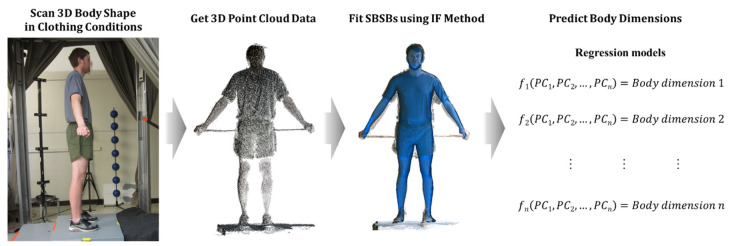
Workflow of the method for prediction of body dimensions from Kinect Scan.

**Figure 4:**
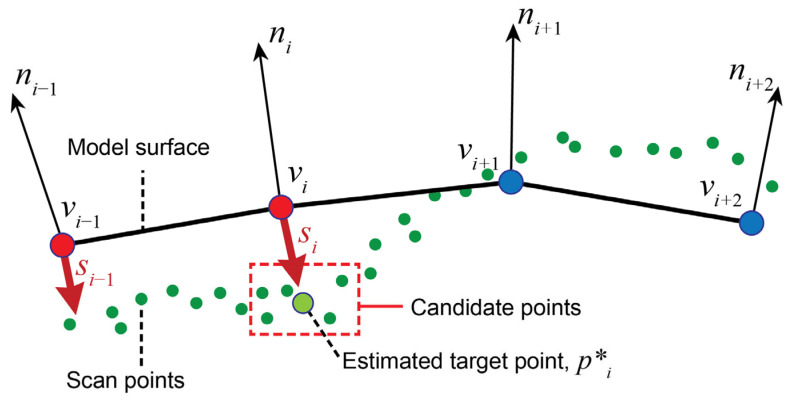
Determination of outside vertices (red) and inscribing vectors toward target points.

In the current study, the IF method was enhanced to deal with noisy point cloud data (PCD) obtained from consumer-grade depth cameras (Figure 3). First, the strategy of the previous IF method to determine the outside vertices was modified due to the absence of the surface normal information in the target PCD. Second, to address noise in the data, we improved the method to find inscribing vectors that move outside vertices to the target surface. Figure 3 shows the improved method to find the inscribing vectors when the target scan is PCD. Let *v_i_* be the *i^th^* vertex of the body shape model and *n_i_* be the normal vector of *v_i_*. To find an inscribing direction for *v_i_*. we first find the closest point *p_i_* among the target points. Since it is possible that *p_i_* is affected by noise and not on the actual target surface, we use an average of ten candidate points that are close to *p_i_* as a modified target point, p*_i_. We determine whether the vertex *v_i_* is outside the target surface (red vertices in Figure 4) by computing the inner product between *n_i_* and *s_i_*, a vector from vi to p*_i_. The ***s*** vectors that have inner product values less than zero are chosen as the inscribing vectors. 

We also improved the fitting algorithm by allowing input of anthropometric constraints, which can improve the validity of estimated body shapes in case of clothed scans. For example, when information such as the individual’s weight and stature is available, we limit the fitting method to find the most feasible body shape in the PC space while keeping the entered stature and weight of the body shape. Regression models were built to associate anthropometric variables with the PC scores, and these regression models were applied at every fitting iteration to adjust the PC scores to meet the targeted anthropometric constraints. If the constraint is before the PC-fitting in each iteration, it can be considered a soft constraint since the PC-fitting can adjust the input constraints. In the current study, we applied a stature constraint only for the clothed (PT) scans.

The final step is to estimate the standardized body dimensions from the PC scores of a fitted avatar. Although a majority of scan-derived anthropometry systems directly measure body dimensions from the mesh of a scan surface, e.g., measuring the geodesic distance between two body landmarks, we rather estimate body dimensions from the PC scores statistically. The main benefit of this approach is that we can estimate dimensions that can be measured from different postures (e.g., seated) using the model. A total of 136 body dimensions available in the MCANSUR dataset were included along with the vertex coordinates in the PCA, so a certain set of PC scores generates the corresponding body dimensions as well as the 3D body shape surface. In this manner, we can obtain the statistically most feasible body dimensions from the PC scores for the underlying body shape the method estimates from a clothed/minimally clad scan. 

### 2.4. Data Collection and Processing

The study protocol was approved by an institutional review board for human-subject research at the University of Michigan (HUM00152937). Volunteers participated in one test session in which body measurements were completed after written informed consent was obtained. We recruited 144 participants (72 women and 72 men) who were approximately representative of the U.S. Marine Corps (USMC) population (Gordon et al., 2013 [3]) with respect to distributions of stature and body mass index. All were between 18 and 35 years of age. The 5th to 95th percentile range in the USMC sample for men’s and women’s stature, 1734 to 1873 mm and 1524 to 1734 mm, respectively, were divided into three stature groups within gender. Within each stature group, the inner 5th to 95th percentile of men’s and women’s BMI, 21.2 to 31.5 kg/m^2^ and 20.2 to 28.1 kg/m^2^, respectively, were divided into higher and lower BMI groups (Table 1).

Table 2 lists the manual anthropometric dimensions obtained in this study while the participants were dressed in minimal clothing, which included bike shorts sized larger than normal to minimize flesh deformation and a sports bra for women. The measurements were intended to be equivalent to those used in the USMC survey (Gordon et al., 2013 [3]). Along with the manual anthropometry, all the participants were scanned in the test posture across the two levels of clothing listed in Table 3.

### 2.5. Statistical Analysis

The goal of the statistical analysis was to validate the method by comparing manually measured anthropometric dimensions with the dimensions predicted from the body scans. The design of the experiment enabled several different evaluations to be performed. The effects of the scan quality were assessed by comparing predictions for both laser scans and Kinect scans for participants in scan wear, which minimized clothing effects. The predictions from Kinect scans in multiple ensembles provided estimates of the effects of clothing on prediction accuracy and precision.

Agreements between the manual measurement and the model-based measurements were assessed using Bland–Altman (B–A) analyses (Bland and Altman, 2007 [20]). B–A plots are often used to compare a new measurement method against a reference method. The difference between the manual (reference) measurement and model-based measurement is plotted as a function of the reference values. The mean (fixed) bias of the model-based measurement was plotted along with the 95% upper and lower limits of agreement (LoA), representing the range of discrepancy within which 95% of new measurements would be expected to lie under the assumption of normality.

## 3. Results

### 3.1. Predictions for Laser Scans in Scan Wear

**Figure 5:**
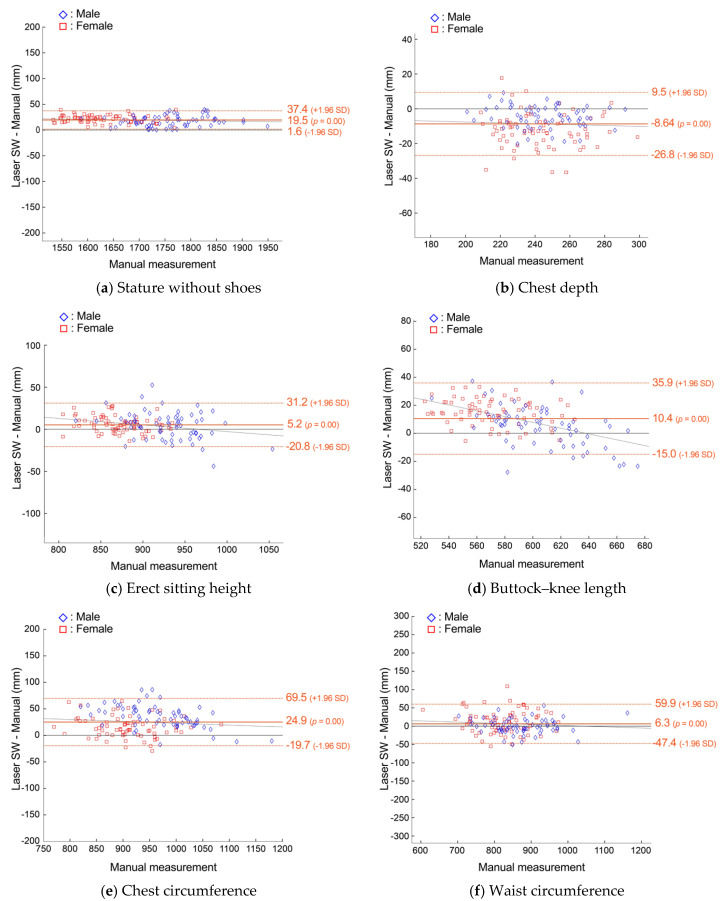
Bland−Altman plots of agreement between manual measurements and predictions from the system on laser scan data with scan wear (SW) clothing for anthropometric dimensions with linear regression lines (black dotted line) and limits of agreement (LoA, 95% prediction limits; orange dotted line).

We first analyzed the anthropometric predictions from the laser scans in SW to evaluate only the developed model-based prediction method without potential errors due to low-resolution and clothed scans. We selected dimensions that show both relatively good and relatively poor performance. Since an essentially infinite number of dimensions could be chosen, we are limiting our reporting to these six for clarity of presentation. Figure 5 shows B–A plots for six dimensions predicted from the male and female scans in scan wear (SW) captured from the whole-body laser scanner. These six dimensions were selected to represent the overall prediction capability of the method against the dimensions that are commonly used in anthropometric surveys and for dimensions that should be measured from other than standing pose, e.g., seated pose. A positive bias of 19.5 mm was observed for stature and erect sitting height and produced the smallest LoA, at ±17.9 mm (1.1% of the mean of manual) and ±26.0 mm (2.9%). Chest depth and waist circumference had the greatest variability, with LoA at ±18.1 mm (7.4%) and ±53.6 mm (6.3%), respectively. The full statistical descriptions of the comparisons are listed in Supplementary Materials.

### 3.2. Predictions for Kinect Scans in Scan Wear

**Figure 6:**
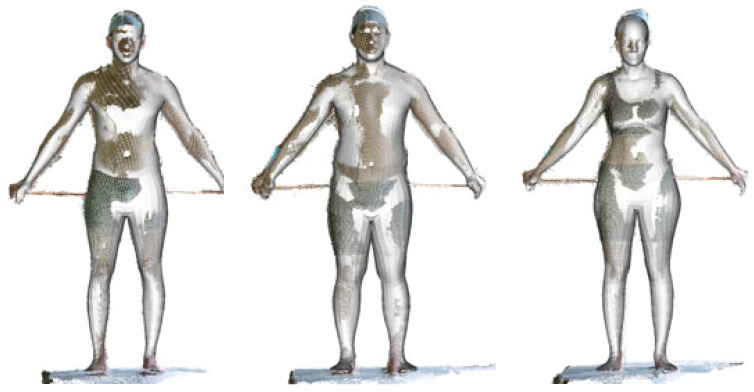
Examples of Kinect scans in SW (in color) and inscribed-fitted manikins (white).

The statistical body shape model was fitted to all the male and female scans in scanwear (SW) captured using the Kinect-based system. Figure 6 shows examples of Kinect scans and the fitted manikins. Rods were used to standardize upper extremity positions. The average fitting time per scan was 690 ms with 50 fitting iterations on a desktop computer (Intel i7 3.6 GHz, 32 GB RAM).

**Figure 7:**
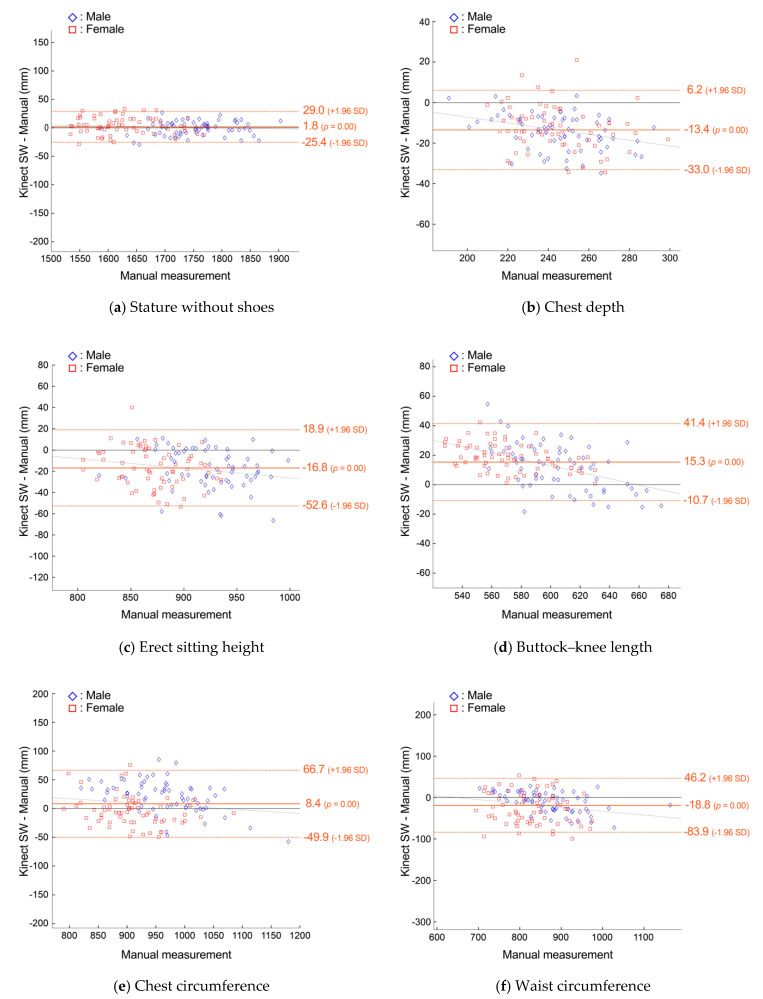
Bland−Altman plots of agreement between manual measurements and predictions from the system on Kinect scan data with scan wear (SW) clothing for anthropometric dimensions with linear regression lines and LoA.

B–A plots in Figure 7 compare the manual measurements and predictions from the Kinect scans in SW. Stature and erect sitting height showed the smallest LoA ranges, at ±27.2 mm (1.6%) and ±35.8 mm (4.0%), while chest depth and waist circumference had the largest percentage LoA, at ±19.6 mm (8.0%) and ±65.1 mm (7.7%), respectively.

### 3.3. Predictions for Kinect Scans in Physical Training (PT) Ensemble

**Figure 8:**
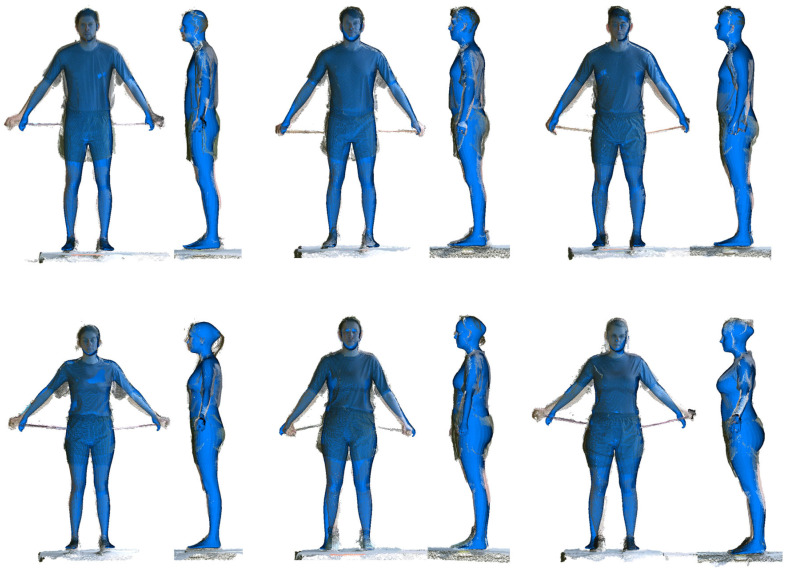
Examples of Kinect scans (point clouds) and inscribed-fitted manikins (blue).

The body shapes were estimated from the scans in the physical training (PT) ensemble using the IF procedure. The average fitting time per scan was 1.1 s. Manually measured statures of the subjects were used as a constraint in the IF procedure. Subject’s stature was used as a fitting constraint to the prediction procedure. Figure 8 shows examples of predicted body shapes under PT ensembles. Qualitatively, the IF method estimated reasonable body shapes from male and female scans that are closely fitted to the exposed skin areas (face, arms, and legs) and lie within the clothing.

**Figure 9:**
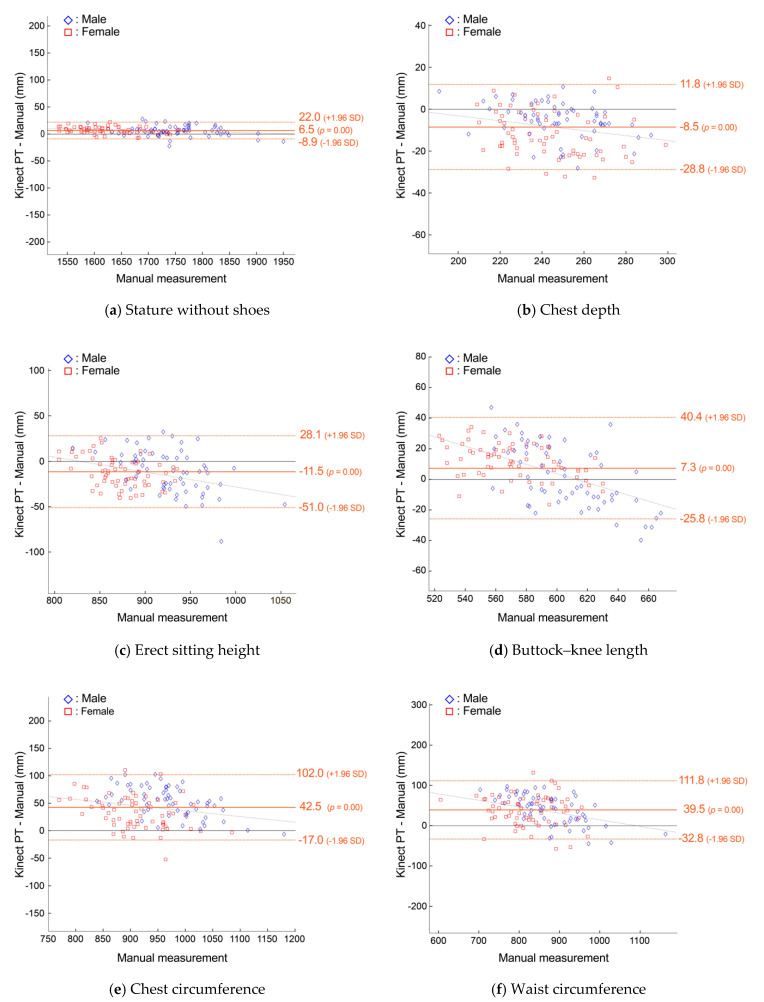
Bland−Altman plots of agreement between manual measurements and predictions from a Kinect-based system with physical training (PT) clothing for anthropometric dimensions with linear regression lines and LoA.

Figure 9 shows that predictions of stature and erect sitting height had the least variability, with LoA at ±15.5 mm (0.9%) and ±39.5 mm (4.4%). As in the other analyses, chest depth and waist circumference demonstrated the largest percentage variability, with LoA at ±20.3 mm (8.3%) and ±72.4 mm (8.6%), respectively.

### 3.4. Comparison of the Predictions across Scanning and Clothing Types

**Figure 10:**
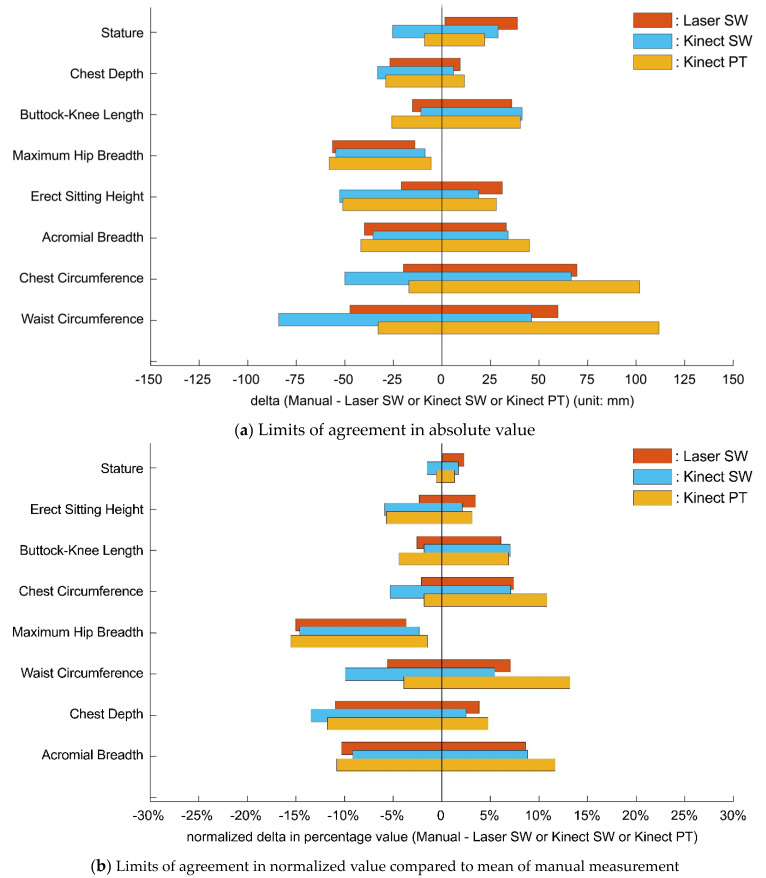
Comparison of the limits of agreement (LoA) range among three methods (Laser SW, Kinect SW, Kinect PT) for each body dimension. Dimensions are vertically arranged with the smallest limits of agreement at the top.

Figure 10 and Table 4 summarize the comparisons of the LoA ranges and mean absolute errors (MAE) across the different systems (laser scanning and Kinect scanning) and different clothing conditions (SW and PT). Figure 10 provides a qualitative view, demonstrating the relative sizes of the LoAs across conditions. Table 4 shows that LoAs for predictions based on Kinect scans were generally larger than for predictions using laser scan data, although for several variables (acromial breadth, chest circumference, and hip breadth), the values were comparable. The difference between scanwear and the PT ensemble was notable only for the circumference measures, for which the LoAs were substantially larger with the PT ensemble.

## 4. Discussion

This study developed and evaluated a model-based anthropometric measurement system using multiple low-resolution cameras and statistical human body shape models (SBSMs). The system estimates realistic underlying body shapes from clothed scans by using the Inscribed Fitting (IF) method. The method was improved in this study to deal with incomplete and noisy point cloud scans obtained from low-cost time-of-flight depth sensors. The new method is designed to be fast, both due to the measurement technology and the fact that the participant does not have to change clothing, and the sensors themselves are low-cost and highly portable when compared to typical scanning systems.

The predicted body dimensions obtained using the new system were compared with actual measurements obtained through the manual method, revealing an overall mean estimation error of 3.3% from SW scans and 3.5% from PT scans. This analysis indicates that the system is somewhat robust to scan quality and light clothing. That is, the distribution of prediction errors is not dramatically affected by using Kinect scans or scanning the participants in clothing. These two findings suggest that the advantages of the system in terms of portability, cost, and measurement speed (no need to change clothing or take a large number of manual measurements) may make it a good choice for certain anthropometric applications. In particular, the system may be most applicable to situations in which a large number of measurements are needed in a short period of time (for example, quantifying the distributions of body dimensions in a difficult-to-measure population) or for applications in which the high accuracy and precision of anthropometry survey grade manual measurements are not as easy to obtain, such as clothing and equipment field evaluations. We note that one of the advantages of the system is that it predicts seated body dimensions without seated body scan data.

This indicates that the prediction performance from clothed scans is comparable with those from minimally clad scans. Bland–Altman analysis indicated minimal bias across predictions except for hip breadth. We note that bias can be readily removed by incorporating either a constant offset or a regression model into the prediction. 

Stature was used as a soft constraint in the predictions for the clothed scans (PT) because this information is generally available in the military application domains on which the research was focused. Predictions without this constraint would be likely to be less accurate, especially for the clothed scans, as shown in Figure 10. High variability around the chest thickness and waist measurement compared to the manual measurements were reported; these may be caused by the influence of clothing, but can also occur due to inconsistency in measuring circumferences for the chest and waist between the training and test datasets, which were measured by different people at different times. Since these dimensions show great variability depending on the measurement locations and methods, errors are also included in the statistical model using these dimensions. The method can make predictions without any constraining manual variables and can handle any number of input variables that are available. In general, adding more data for the body dimension prediction from the participant is useful, but the benefit of incorporating more values than sex, stature, body weight (or BMI), and erect sitting height is minimal. 

The major limitation of the IF method is that it is sensitive to the clothing type since it estimates the body shape fully based on the clothing surface. If the clothing and the equipment surfaces provide less information about the body shape (e.g., astronaut in a space suit), the estimation accuracy is likely to be lower. Also, as with all whole-body scanning methods, this system cannot provide accurate estimates of body dimensions for which minimal data is available in the scan. In particular, obtaining accurate head, hands, and feet dimensions would require scan data focused on those regions. For these variables, the system outputs are plausible but tend toward the mean, reflecting the relatively weak correlations between these dimensions and overall body size. However, although the errors tend to be larger than allowable errors presented in other reports, such as ANSUR or ISO-20685-1 [21], this is a reasonable trade-off in some practical applications for the speed and efficiency gains from measuring clothed individuals. We note that few manual measurement programs, particularly those related to clothing, approach the allowable error values, which are based on highly trained individuals making time-consuming measurements on minimally clad individuals.

These results are also limited by the relatively lean study population, which was chosen to be approximately representative of the military population the model was trained on. Although the system can function for any population, the results will be most accurate when the study population is similar to the training data with respect to size and shape. Future work will include adding more clothed and minimally clad scan data of a wide range of individuals and training the system using machine-learning techniques, not only to improve the prediction accuracy but also to automatically characterize clothing ensembles and enhance torso-related body dimensions (e.g., chest circumference, waist circumference) for improved accuracy.

## 5. Conclusions

In this current study, we presented and validated a model-based anthropometric system utilizing low-resolution and low-cost sensors in conjunction with a statistical body shape model known as the SBSM. The newly enhanced inscribed fitting method enables estimation of body shape and dimensions from an incomplete and noisy scan of a lightly clothed individual. This overcomes the limitations of traditional scan-derived anthropometry methods by allowing substantially reduced measurement time/effort and providing repeatable measurements of standardized body dimensions. Our method demonstrated flexibility and adaptability in complex anthropometric measurements across various domains—ranging from healthcare to fashion and personalized equipment design.

## Figures and Tables

**Table 1:** Participant size distribution.

**Gender**	**Stature Percentile**	**Stature (mm)**		**Sample Size (*n*)**		
				**BMI: 21–24**	**BMI: 25–28**	**Total**
F	5–25th	1524	1581	12	6	18
F	25th–75th	1581	1664	24	12	36
F	75th–95th	1664	1734	12	6	18
				48	24	72
**Gender**	**Stature Percentile**	**Stature (mm)**		**Sample Size (*n*)**		
				**BMI: 21–26**	**BMI: 27–31**	**Total**
M	5–25th	1647	1707	12	6	18
M	25th–75th	1707	1797	24	12	36
M	75th–95th	1797	1873	12	6	18
				48	24	72

**Table 2:** List of standard anthropometric dimensions.

Posture	Body Dimensions	
Standing	Weight	Chest depth (flat blades, max anterior pt)
	Stature without shoes	Height of chest (max anterior pt)
	Eye height	Waist circumference at omphalion (ANSUR protocol)
	Acromial height	Waist height at omphalion (ANSUR protocol)
	Cervical height	Hip circumference at buttocks (ANSUR protocol)
	Acromial breadth	Tragion to top of head
	Bideltoid breadth	Head length
	Bicristal breadth	Head breadth
	Acromion—radiale length	Hand length
	Radiale—stylion length	Hand breadth
	Lower arm length (elbow–fingertip)	Thigh length: top of trochanter to lateral epicondyle along thigh
	Upper arm length (acromion to olacronon)	Shank length: from medial condyle to medial malleolus along shank
	Chest breadth (max anterior pt)	
	Chest circumference (max anterior pt)	
Sitting	Erect sitting height	Knee height
	Buttock–knee length	Popliteal height
	Buttock–popliteal length	Maximum hip breadth

**Table 3:** Levels of clothing.

Body Region	Scan Wear (SW)	Physical Training (PT)
Feet	Bare feet	Participant’s exercise shoes
Lower	Modified compression shorts worn a size larger	Nylon tricot running shorts with 5” inseam over compression shorts
Top	Men: nothing; women: sports bra	Moisture wicking T-shirt
Head	Men: no hair cap; women: elastic hair cap	

Note: Example images categorized by levels of clothing are displayed in Figure 2.

**Table 4:** Analysis results of limits of agreement (LoA, mm) among three methods (Laser SW, Kinect SW, Kinect PT) for each body dimension.

Limits of Agreement (LoA)	Laser SW	Kinect SW	Kinect PT
Stature without shoes	19.5	11.2	8.4
Chest depth	10.0	14.4	10.6
Buttock−knee length	14.2	17.5	15.7
Maximum hip breadth	34.9	31.6	31.9
Erect sitting height	11.1	19.7	18.7
Acromial breadth	15.2	14.7	17.2
Chest circumference	27.7	24.4	44.2
Waist circumference	21.5	30.3	46.1

## Data Availability

This is an ongoing study, and the results will be available to the public on Humanshape.org upon completion.

## Supplementary Materials

The following supporting information can be downloaded at: https://www.mdpi.com/article/10.3390/s24051350/s1, Table S1: Descriptive statistics of anthropometric dimensions for Manual measurement and Predictions from the fitted Statistical Body Shape Models (SBSMs) on Laser scan with scan wear (SW) clothing; Table S2: Descriptive statistics of anthropometric dimensions for Manual measurement and Predictions from the Kinect-based system on Laser scan with scan wear (SW) clothing; Table S3: Descriptive statistics of anthropometric dimensions for Manual measurement and Predictions from the Kinect-based system on Laser scan with physical training (PT) clothing; Table S4: Comparison of mean bias and limits of agreement (LoA) among three methods (Laser SW, Kinect SW, Kinect PT) for body dimensions.

## References

[1] Bartol K., Bojanić D., Petković T., Pribanić T. (2021). A Review of Body Measurement Using 3D Scanning. IEEE Access.

[2] Bonin D., Ackermann A., Radke D., Peters M., Wischniewski S. (2023). Anthropometric Dataset for the German Working-Age Population Using 3D Body Scans from a Regional Epidemiological Health Study and a Weighting Algorithm. Ergonomics.

[3] Gordon C.C., Blackwell C.L., Bradtmiller B., Parham J.L., Hotzman J. (2013). 2010 Anthropometric Survey of U.S. Marine Corps Personnel: Methods and Summary Statistics.

[4] Gordon C.C., Blackwell C.L., Bradtmiller B., Parham J.L., Barrientos P., Paquette S.P., Corner B.D., Carson J.M., Venezia J.C., Rockwell B.M. (2014). 2012 Anthropometric Survey of U.S. Army Personnel: Methods and Summary Statistics.

[5] Goto L., Lee W., Molenbroek J.F.M., Cabo A.J., Goossens R.H.M. (2019). Traditional and 3D Scan Extracted Measurements of the Heads and Faces of Dutch Children. Int. J. Ind. Ergon..

[6] Lu J.M., Wang M.J.J. (2008). Automated Anthropometric Data Collection Using 3D Whole Body Scanners. Exp. Syst. Appl..

[7] Park B.-K., Lumeng J.C., Lumeng C.N., Ebert S.M., Reed M.P. (2014). Child Body Shape Measurement Using Depth Cameras and a Statistical Body Shape Model. Ergonomics.

[8] Robinette K.M., Blackwell S., Daanen H., Boehmer M., Fleming S., Brill T., Hoeferlin D., Burnsides D. (2002). Civilian American and European Surface Anthropometry Resource (CAESAR).

[9] Tsoli A., Loper M., Black M. Model-based Anthropometry: Predicting Measurements from 3D Human Scans in Multiple Poses. Proceedings of the 2014 IEEE Winter Conference on Applications of Computer Vision (WACV 2014).

[10] Guan P., Freifeld O., Black M.J. (2010). A 2D Human Body Model Dressed in Eigen Clothing. Computer Vision—ECCV 2010.

[11] Hasler N., Rosenhahn B., Thormählen T., Stoll C. (2009). Estimating Body Shape of Dressed Humans. Comput. Graph..

[12] Hu P., Kaashki N.N., Dadarlat V., Munteanu A. (2021). Learning to Estimate the Body Shape under Clothing from a Single 3-D Scan. IEEE Trans. Ind. Inform..

[13] Pishchulin L., Wuhrer S., Helten T., Theobalt C., Schiele B. (2017). Building Statistical Shape Spaces for 3D Human Modeling. Pattern Recognit..

[14] Yang J., Franco J.S., Hétroy-Wheeler F., Wuhrer S. (2016). Estimation of Human Body Shape in Motion with Wide Clothing. Computer Vision—ECCV 2016.

[15] Lu Y., Cha J.-H., Youm S.-K., Jung S.-W. (2021). Parametric Shape Estimation of Human Body Under Wide Clothing. IEEE Trans. Multimed..

[16] Bălan A.O., Black M.J. (2008). The Naked Truth: Estimating Body Shape Under Clothing. Computer Vision—ECCV 2008.

[17] Zhang C., Pujades S., Black M.J., Pons-Moll G. Detailed, Accurate, Human Shape Estimation from Clothed 3D Scan Sequences. Proceedings of the 2017 IEEE Conference on Computer Vision and Pattern Recognition.

[18] Park B.-K.D., Corner B.D., Kearney M., Reed M.P. Estimating Human Body Characteristics under Clothing Using a Statistical Body Shape Model. Proceedings of the 4th International Digital Human Modeling Conference.

[19] Park B.-K., Reed M.P. (2015). Parametric Body Shape Model of Standing Children Ages 3 to 11 Years. Ergonomics.

[20] Bland J.M., Altman D.G. (2007). Agreement between Methods of Measurement with Multiple Observations per Individual. J. Biopharm. Stat..

[21] (2018). 3-D Scanning Methodologies for Internationally Compatible Anthropometric Databases Part 1: Evaluation Protocol for Body Dimensions Extracted from 3-D Body Scans.

