# Supplementary material for: Efficient Model-Based Anthropometry under Clothing Using Low-Cost Depth Sensors
Source: Sensors (Basel). 2024 Feb 20;24(5):1350. doi: 10.3390/s24051350 (PMC10934441; doi:10.3390/s24051350)
Supplement: Supplementary file 1 [file sensors-24-01350-s001.zip › sensors-2854143-supplementary.pdf]

sensors

MDPI

Supplementary Tables

Table S1. Descriptive statistics of anthropometric dimensions for Manual measurement and Predictions from the fitted Statistical Body Shape Models (SBSMs) on Laser scan with scan wear (SW) clothing (Unit: mm, sample size (n): 136)

Methods		Stature without Shoes	BMI (Kg/m²)	Erect Sitting Height	Acromial Breadth	Buttock-Knee Length	Chest Circumfe-rence	Chest Depth	Maximum Hip Breadth	Waist Circumfe-rence
Manual	Mean	1689.5	24.1	898.4	385.9	587.1	935.7	243.9	373.9	845.4
	SD	94.0	2.7	45.6	29.6	34.0	70.9	20.0	24.1	79.6
	5th	1550.3	20.2	821.2	340.6	535.3	820.0	214.3	335.0	724.3
	10th	1564.1	21.0	842.1	349.1	543.1	849.1	220.0	344.0	735.5
	25th	1606.5	22.1	864.5	364.0	562.0	890.0	227.5	358.5	795.5
	50th	1690.0	24.0	893.0	382.0	582.0	928.0	242.0	373.0	842.5
	75th	1758.5	26.3	932.0	406.5	611.5	986.0	258.5	388.5	890.5
	90th	1823.9	27.4	962.7	430.9	630.9	1027.9	270.0	406.8	945.0
	95th	1846.7	29.0	969.7	439.1	652.0	1050.3	279.7	413.7	971.4
Laser SW	Mean	1709.0	23.8	903.6	382.6	597.6	960.6	235.2	339.0	851.7
	SD	93.2	2.6	43.9	34.9	29.1	72.1	21.6	20.8	81.7
	5th	1571.7	19.6	836.2	332.1	547.7	850.0	201.5	306.3	729.8
	10th	1588.3	20.9	845.4	339.0	558.9	870.4	207.2	312.2	748.1
	25th	1628.3	22.0	869.7	352.4	576.1	906.6	219.1	325.6	799.1
	50th	1711.9	23.7	899.9	378.1	595.2	954.6	233.3	336.9	844.0
	75th	1776.4	25.6	937.7	415.2	618.5	1017.4	251.5	353.8	910.3
	90th	1848.4	27.3	958.3	426.1	637.1	1052.1	263.7	362.7	962.5
	95th	1869.6	28.3	976.8	436.2	644.7	1068.3	273.1	374.8	983.9

Table S2. Descriptive statistics of anthropometric dimensions for Manual measurement and Predictions from the Kinect-based system on Laser scan with scan wear (SW) clothing (Unit: mm, sample size (*n*): 129)

Methods		Stature without Shoes	BMI (Kg/m²)	Erect Sitting Height	Acromial Breadth	Buttock-Knee Length	Chest Circumfe-rence	Chest Depth	Maximum Hip Breadth	Waist Circumfe-rence
Manual	Mean	1689.1	24.3	897.3	385.9	588.2	936.8	244.4	373.8	848.2
	SD	88.5	2.7	42.7	29.1	32.7	68.6	20.2	23.6	78.2
	5th	1550.0	20.2	823.8	341.8	538.0	828.6	215.0	334.9	725.0
	10th	1568.2	21.0	842.4	349.4	549.0	855.0	220.0	344.0	740.0
	25th	1611.5	22.2	865.8	364.8	562.0	890.0	228.0	360.0	795.8
	50th	1694.0	24.3	893.0	382.0	582.0	930.0	243.0	373.0	843.0
	75th	1755.5	26.2	930.3	406.0	613.3	982.8	257.0	389.3	893.5
	90th	1815.6	27.7	958.2	427.4	630.0	1025.4	270.0	406.2	952.2
	95th	1834.3	29.1	969.0	434.3	652.0	1044.5	283.0	411.1	972.0
Kinect SW	Mean	1690.9	23.6	880.5	385.3	603.5	945.1	230.9	342.1	829.4
	SD	88.1	2.7	42.8	29.2	28.0	70.6	19.8	20.2	77.9
	5th	1557.7	19.5	815.0	341.0	558.4	836.3	198.9	305.3	711.0
	10th	1570.7	20.2	818.1	347.1	567.5	857.2	207.6	316.7	734.6
	25th	1616.5	21.5	850.3	359.7	583.1	893.6	216.7	328.3	772.6
	50th	1699.3	23.4	880.1	383.0	601.4	933.3	229.5	341.8	827.0
	75th	1756.6	25.5	910.9	409.5	625.4	1004.1	242.2	356.8	882.2
	90th	1813.2	26.9	934.5	425.5	640.5	1036.7	257.4	366.8	922.7
	95th	1839.4	28.8	949.7	429.3	651.7	1065.1	265.2	374.8	949.3

Table S3. Descriptive statistics of anthropometric dimensions for Manual measurement and Predictions from the Kinect-based system on Laser scan with physical training (PT) clothing (Unit: mm, sample size (*n*): 127).

Methods		Stature without Shoes	BMI (Kg/m²)	Erect Sitting Height	Acromial Breadth	Buttock-Knee Length	Chest Circumfe-rence	Chest Depth	Maximum Hip Breadth	Waist Circumfe-rence
Manual	Mean	1688.5	24.3	897.2	385.8	588.1	938.2	245.4	374.1	847.4
	SD	89.7	2.7	44.8	29.0	32.9	70.1	20.4	24.2	80.7
	5th	1549.9	20.4	820.0	341.7	536.9	819.9	214.6	336.7	722.8
	10th	1575.2	21.0	842.2	349.2	544.2	855.0	220.2	344.0	742.0
	25th	1613.5	22.3	864.0	365.0	564.3	891.3	229.3	358.3	795.0
	50th	1692.0	24.5	892.0	382.0	586.0	934.0	245.0	372.0	843.0
	75th	1752.3	26.2	929.5	405.0	613.0	977.8	260.8	388.0	896.5
	90th	1816.8	27.6	959.6	429.8	630.8	1031.2	270.0	407.0	947.4
	95th	1834.9	29.1	969.0	434.5	652.0	1054.8	283.0	416.2	970.3
Kinect PT	Mean	1695.0	23.1	885.8	387.5	593.9	975.5	235.1	340.7	880.6
	SD	88.3	4.2	42.0	34.2	32.2	92.4	28.2	26.5	97.6
	5th	1556.6	19.6	819.8	335.4	550.1	875.2	203.3	304.5	767.8
	10th	1583.0	20.1	834.1	345.9	557.0	888.8	211.2	316.4	790.1
	25th	1618.9	21.6	853.1	358.6	578.1	925.9	222.4	328.4	832.4
	50th	1698.3	23.3	884.9	381.6	598.3	977.0	235.2	340.2	883.3
	75th	1756.6	25.1	913.2	417.8	614.2	1032.3	250.1	355.7	943.3
	90th	1821.6	26.3	947.1	431.7	627.1	1067.5	263.7	366.2	984.1
	95th	1844.5	27.8	958.0	438.0	638.5	1091.1	273.7	375.8	995.5

Table S4. Comparison of mean bias and limits of agreement (LoA, mm) among three methods (Laser SW, Kinect SW, Kinect PT) for body dimensions (Unit: mm)

Dimensions	Manual		Manual vs. Laser SW		Manual vs. Kinect SW		Manual vs. Kinect PT	
	Mean	SD	Bias	LoA (% of mean manual)	Bias	LoA (% of mean manual)	Bias	LoA (% of mean manual)
Stature w/o shoes	1691.4	91.8	19.5	±18.0 (1.1%)	1.1	±31.9 (1.9%)	6.6	±12.4 (0.7%)
Cervicale Height	1460.5	83.3	4.7	±26.5 (1.8%)	-4.4	±34.6 (2.4%)	-1.0	±25.0 (1.7%)
Chest Height	1250.4	77.1	-12.2	±41.1 (3.3%)	-19.6	±42.8 (3.4%)	-21.0	±46.9 (3.8%)
Weight w/o shoes(Kg)	69.6	11.6	0.8	±3.8 (5.5%)	-1.6	±5.3 (7.6%)	-2.7	±19.8 (28.5%)
Erect Sitting Height	898.8	44.3	5.3	±26.1 (2.9%)	-17.5	±37.5 (4.2%)	-11.1	±37.5 (4.2%)
Eye Height (Sitting)	789.8	42.2	1.5	±36.9 (4.7%)	-16.7	±41.5 (5.3%)	-12.0	±39.9 (5.1%)
Knee Height	515.4	33.2	21.3	±22.0 (4.3%)	21.4	±26.6 (5.2%)	20.0	±27.8 (5.4%)
Popliteal Height	419.0	32.5	-10.1	±35.5 (8.5%)	-5.4	±34.7 (8.3%)	-2.2	±36.0 (8.6%)
Head Length	192.8	8.4	8.1	±11.1 (5.7%)	0.4	±15.5 (8.0%)	1.1	±19.2 (9.9%)
Head Breadth	154.6	6.6	-4.9	±9.0 (5.8%)	-10.3	±13.6 (8.8%)	-9.4	±15.9 (10.3%)
Tragion to Top of Head	125.5	8.0	5.6	±14.6 (11.6%)	1.04	±17.3 (13.8%)	3.1	±16.2 (12.9%)
Buttock-Knee Length	588.3	33.5	10.4	±25.6 (4.3%)	15.3	±25.9 (4.4%)	5.5	±47.0 (8.0%)
Buttock-Popliteal Length	483.6	30.4	1.9	±31.7 (6.6%)	9.4	±32.3 (6.7%)	2.3	±42.6 (8.8%)
Acromial Breadth	386.7	29.1	-3.4	±36.6 (9.5%)	-0.7	±35.5 (9.2%)	1.2	±43.2 (11.2%)
Chest Breadth	281.5	25.8	-12.5	±38.1 (13.5%)	-13.7	±38.5 (13.7%)	-1.7	±41.1 (14.6%)
Chest Depth (scapula)	244.2	20.0	-8.7	±18.2 (7.5%)	-13.7	±20.0 (8.2%)	-10.5	±45.7 (18.7%)
Bi-Cristal Breadth	296.1	25.2	-30.7	±33.3 (11.3%)	-34.3	±38.6 (13.0%)	-21.9	±42.8 (14.4%)
Upper Arm Length	355.4	23.8	-4.2	±20.7 (5.8%)	-7.4	±26.2 (7.4%)	-5.0	±24.9 (7.0%)
Lower Arm Length	460.6	35.1	-7.2	±47.1 (10.2%)	-12.8	±48.8 (10.6%)	-13.7	±31.8 (6.9%)
Hand Length	186.5	11.8	-1.2	±18.3 (9.8%)	-6.1	±19.8 (10.6%)	-5.9	±17.9 (9.6%)
Hand Breadth	79.1	5.8	2.9	±7.9 (10.0%)	1.4	±11.4 (14.4%)	0.1	±11.0 (14.0%)

**Dimensions**	**Manual**		**Manual vs. Laser SW**		**Manual vs. Kinect SW**		**Manual vs. Kinect PT**	
	**Mean**	**SD**	**Bias**	**LoA (% of mean manual)**	**Bias**	**LoA (% of mean manual)**	**Bias**	**LoA (% of mean manual)**
Acromion-Radiale Length	317.7	21.4	4.5	±19.1 (6.0%)	4.5	±22.6 (7.1%)	6.7	±22.7 (7.1%)
Radiale-Stylion Length	246.8	19.9	5.7	±31.4 (12.7%)	4.3	±24.1 (9.7%)	1.4	±26.2 (10.6%)
Thigh Length	431.7	29.8	-24.2	±48.3 (11.2%)	-24.0	±44.6 (10.3%)	-31.9	±45.0 (10.4%)
Shank Length	367.5	32.4	41.3	±43.1 (11.7%)	45.8	±44.3 (12.0%)	48.9	±44.5 (12.1%)
Chest Circumference	937.2	68.8	24.9	±44.8 (4.8%)	8.3	±58.1 (6.2%)	36.2	±130.5 (13.9%)
Waist Circumference (omph)	847.7	78.5	5.9	±53.2 (6.3%)	-18.5	±65.4 (7.7%)	32.9	±158.8 (18.7%)
Waist Circumference Height	1012.9	64.8	22.6	±42.1 (4.2%)	12.1	±40.6 (4.0%)	7.3	±43.5 (4.3%)
Hip Circumference	984.4	58.1	9.4	±32.7 (3.3%)	21.8	±35.8 (3.6%)	13.3	±127.4 (12.9%)
